# Tri-Trophic Insecticidal Effects of African Plants against Cabbage Pests

**DOI:** 10.1371/journal.pone.0078651

**Published:** 2013-10-24

**Authors:** Blankson W. Amoabeng, Geoff M. Gurr, Catherine W. Gitau, Helen I. Nicol, Louis Munyakazi, Phil C. Stevenson

**Affiliations:** 1 School of Agriculture and Wine Sciences, Charles Sturt University, Orange Campus, Orange, New South Wales, Australia; 2 E. H. Graham Centre for Agricultural Innovation (NSW Department of Primary Industries and Charles Sturt University), Orange, New South Wales, Australia; 3 Department of Mathematics and Statistics, Kumasi Polytechnic, Kumasi, Ghana; 4 Natural Resources Institute, University of Greenwich, Kent, United Kingdom; 5 Royal Botanic Gardens, Kew, Surrey, United Kingdom; 6 Council for Scientific and Industrial Research (CSIR) - Crops Research Institute, Kumasi, Ghana; French National Institute for Agricultural Research (INRA), France

## Abstract

Botanical insecticides are increasingly attracting research attention as they offer novel modes of action that may provide effective control of pests that have already developed resistance to conventional insecticides. They potentially offer cost-effective pest control to smallholder farmers in developing countries if highly active extracts can be prepared simply from readily available plants. Field cage and open field experiments were conducted to evaluate the insecticidal potential of nine common Ghanaian plants: goat weed, *Ageratum conyzoides* (Asteraceae), Siam weed, *Chromolaena odorata* (Asteraceae), Cinderella weed, *Synedrella nodiflora* (Asteraceae), chili pepper*, Capsicum frutescens* (Solanaceae), tobacco, *Nicotiana tabacum* (Solanaceae) cassia, *Cassia sophera* (Leguminosae), physic nut, *Jatropha curcas* (Euphorbiaceae), castor oil plant, *Ricinus communis* (Euphorbiaceae) and basil, *Ocimum gratissimum* (Lamiaceae). In field cage experiments, simple detergent and water extracts of all botanical treatments gave control of cabbage aphid, *Brevicoryne brassicae* and diamondback moth, *Plutella xylostella*, equivalent to the synthetic insecticide Attack^®^ (emamectin benzoate) and superior to water or detergent solution. In open field experiments in the major and minor rainy seasons using a sub-set of plant extracts (*A. conyzoides, C. odorata*, *S.* nodiflora*, N. tabacum* and *R. communis*), all controlled *B. brassicae* and *P. xylostella* more effectively than water control and comparably with or better than Attack^®^. Botanical and water control treatments were more benign to third trophic level predators than Attack^®^. Effects cascaded to the first trophic level with all botanical treatments giving cabbage head weights, comparable to Attack^®^ in the minor season. In the major season, *R. communis* and *A conyzoides* treatment gave lower head yields than Attack^®^ but the remaining botanicals were equivalent or superior to this synthetic insecticide. Simply-prepared extracts from readily-available Ghanaian plants give beneficial, tri-trophic benefits and merit further research as an inexpensive plant protection strategy for smallholder farmers in West Africa.

## Introduction

Cabbage, *Brassica oleracea* var. *capitata* L. (Cruciferae) is an important temperate vegetable crop that grows well in other climatic regions throughout the world [1]. Despite the importance of cabbage, there are a number of biotic constraints, including insect pests, which hamper its production and consumption [2]. 

 Diamondback moth (DBM), *Plutella xylostella* (L.) (Lepidoptera: Plutellidae) is of European origin and one of the most destructive pest of crucifers worldwide [3,4]. Yield loss attributed to DBM can be as high as 100% [2,5]. Global cost of control and yield loss attributed to DBM has recently been estimated between US$ 4 and 5 billion per annum [6,7]. In Ghana, DBM is considered the most important pest of cabbage [8].

The cabbage aphid, *Brevicoryne brassicae* (L.) (Hemiptera: Aphididae) is also an important insect pest of cabbage in Ghana and globally [9]. The four nymphal stages and the adult aphid are phloem feeders [10]. Their feeding results in weak, wrinkled leaves that are cupped both outward and inward, resulting in a deformed plant with lower yields [9,10]. Indirect damage from their feeding result from the excreta (honeydew) that supports the growth of sooty mould [10]. In addition, the cabbage aphid is a vector of 23 virus diseases of Cruciferae [11]. 

Though both of these pests are attacked by a range of natural enemy species, biological control is rarely considered adequate; to the extent that in Ghana, other parts of Africa, and globally, vegetable growers frequently apply synthetic insecticides to manage DBM, cabbage aphid and other prevalent insect pests of cabbage [6,8,12-15]. Synthetic insecticides work relatively quickly, are easy to apply and are not labour intensive [5]. There has, however, been an increase in the resistance of DBM and other insect pests of cabbage to insecticides, making their management difficult [4,17]). Synthetic insecticides have also been associated with health hazards to humans and animals, environmental pollution, pest resistance, and are unavailable to many peasant farmers such as those in the Ghanaian hinterlands [16,17]). Synthetic insecticides are often mishandled and misapplied especially by inexperienced farmers [13,18]). In order to avoid the negative impacts of these synthetic insecticides, alternative approaches to managing pests of cabbage and other vegetables must be sought [13,18]). Generally, the use of botanical insecticides is more sustainable and has a lower environmental impact than synthetic insecticides [16,19-22]). However, current commercially extracted botanical insecticides such as pyrethrum and azadirachtin tend to be relatively expensive and difficult for most smallholder farmers to obtain. To constitute a viable technology for most of the world’s poor farmers, botanical insecticides must be based on plant materials that are cheap and readily available and be simply prepared rather than requiring organic solvents and complex apparatus. Further, extracts need to be benign to natural enemies in order to avoid secondary and resurgent pests, as well as having low phytotoxicity and protecting yields. 

Though simple plant extracts are commonly promoted for use in home gardens, there is growing interest in their potential for farmers in developing countries. In Ghana, for example, chili *Capsicum frutescens* (Solanaceae) extract concentrations of 15, 20 and 30g/L of water gave a significant reduction in *B. brassicae* numbers compared to λ-cyhalothrin [23]. Other work, in Uganda, demonstrated that crude aqueous extracts of tobacco, *Nicotiana tabacum* (Solanaceae) and *Tephrosia* sp. (Fabaceae) were as efficacious as the synthetic insecticides, Cypermethrin^®^ and Fenitrothion^®^ in reducing emergence of bruchid beetle, *Callosobruchus* sp. [24] Similarly, in Nigeria, extracts of garlic, *Allium sativum* (Asparagales: Amaryllidaceae) chili pepper, ginger, *Zingiber officinale* (Zingiberales: Zingiberaceae) neem, *Azadirachta indica* (Sapindales: Meliaceae) tobacco and sweetsop, *Annona squamosa* (Magnoliales: Annonaceae) have been successfully used to control pests of cowpea [25]. In Ghana, crude leaf and seed extracts of neem tree have been used extensively in managing pests of crops such as cabbage, lettuce and cowpea [4,9]. 

Pest management using local materials offers farmers the opportunity to reduce production costs, as the plants often grow wild in and around farms so can be obtained with little effort and zero or minimal cost. In addition, cultivation of insecticidal plant species that might otherwise be locally scarce represents scope for agricultural diversification and complementary income sources. Some plant species with potentially useful insecticidal properties in the families of Meliaceae, Rutaceae, Asteraceae, Piperaceae, Compositae, Lamiaceae, Euphorbiaceae, Combretaceae and Annonaceae are common weed, shrub and tree species on and around farms [16]. 

Given the need for alternatives to conventional insecticides and the potential utility of extracts from locally-growing plants, the aim of the current study was to identify from amongst plants that are common in Ghana those with utility against cabbage pests. A field cage experiment was conducted to screen crude water plus detergent extracts of nine such plants against *P. xylostella* and *B. brassicae*. Five of these plants were then trialed in two field experiments conducted in the major and minor rainy seasons. Pest incidence usually varies between the two rainy seasons, higher in minor than the major rainy season. It was therefore important to test the treatments in both seasons in order to make a full assessment of their performance against the pests.

## Materials and Methods

### Study site

Experiments were conducted at the Council for Scientific and Industrial Research (CSIR)-Crops Research Institute (CRI), Kwadaso, Kumasi, Ghana (Latitude 6°43'N Longitude 1°36'W; 287m elevation). The field cage experiment was carried out between January and April whilst the open field experiments were between May and August and July and October, 2012 for major and minor rainy seasons, respectively. The study site was part of the wet semi-deciduous forest ecological zone of Ghana with annual rainfall between 1200 and 1600mm. Mean minimum and maximum ambient temperature during both experiments ranged between 22-31°C, with the mean relative humidity ranging from 75-78% and 78-82% for the field cage and open field experiments, respectively.

### Experimental design and treatment preparation

#### Field cage experiments

*Plutella xylostella* and *B. brassicae* were collected from local cabbage fields and cultured in cages containing potted cabbages. Permission to collect the initial *P. xylostella* and *B. brassicae* for the laboratory culture was granted by the local farmer. Multiple cages were used for each species so that any affected by disease or parasitism could be quarantined from further use. The two herbivores were tested against each treatment in separate experiments. For each experiment, treatments consisted of four controls and nine botanical treatments and were set up in a randomised complete block design with three replications. Positive control treatments were the two synthetic insecticides, Attack^®^ (emamectin benzoate) and Lambda Super^®^ 2.5 EC (lambda-cyhalothrin), whilst negative control treatments were 0.1% Sunlight^®^ detergent solution and tap water. Attack^®^ and Lambda super^®^ were applied at the recommended rates of 1.5mL/L and 4.8mL/L of water, respectively. The nine botanical treatments were prepared from each of the following plant species; goat weed, *Ageratum conyzoides*, Siam weed, *Chromolaena odorata*, Cinderella weed, *Synedrella nodiflora*, hot pepper*, Capsicum frutescens*, tobacco, *Nicotiana tabacum Cassia*, *Cassia sophera*, physic nut, *Jatropha curcas*, castor oil plant, *Ricinus communis* and basil, *Ocimum gratissimum*. For most plant species fresh leaves were collected from within 1km of the experimental site and 30g fresh weight of each was pounded into a pulp in a wooden mortar using a wooden pestle. Reflecting the fact that plant chemistry can vary between individuals, the plant materials for this study were collected from several individual plants from more than five different locations and were thoroughly mixed before the required quantities were taken as a sub-sample to prepare the extracts. The plant identifications were confirmed by a botanist prior to the preparation of the extracts. Voucher specimens of all the plant species were deposited at the herbarium of the National Genebank, of CSIR- Plant Genetic Resource Research Institute, Bunso, Eastern region, Ghana for future study. The mortar and pestle were washed with a sponge and detergent and given multiple rinses of tap water after each plant material. For *C. frutescens*, ripe fruits were obtained from a local market and homogenised using an electric blender. Processed plant materials were each mixed with 1L tap water containing 0.1% Sunlight^®^ detergent solution to give a 3% w/v final concentration then sieved through fine linen into a 2L capacity hand sprayer for immediate application.

For each experiment, a 38-day-old potted cabbage plant (cv. Oxylus) with six true leaves was covered with an insect-proof net fitted with an elastic band at the base and a zipper at the side to enable access. Potted plants were covered with the net immediately after potting. Seedlings for potting were raised on a seed bed that was completely covered with insect proof net from day of sowing till seedlings were potted. Plants were arranged 60cm apart on a one metre high wooden platform. For the *P. xylostella* experiment, plants were infested with 10 second generation (from field collection); third instar larvae using a fine brush. Larvae were allowed to feed for three days by which time they had reached fourth instar larval stage before the application of treatments. For the *B. brassicae* experiment, 20 third generation adult cabbage aphids were transferred onto the plants using a fine camel hair brush and were allowed to establish colonies for seven days before treatment. All treatments were applied to the point of runoff to infested potted plants through a zipper at the side of the cage. Only one treatment application was done in *P. xylostella* experiment whilst two treatment applications were done in the *B. brassicae* experiment with a seven day interval between the two applications.

Numbers of *P. xylostella* larvae were assessed 48 hours after spraying and percentage mortality calculated. *Brevicoryne brassicae* were much more numerous and difficult to count without disruption so were scored using a modified method of [26] as: 0 = absent, 1 = a few scattered individuals, 2 = a few isolated small colonies, 3 = several small isolated colonies, 4 = large isolated colonies, 5 = large continuous colonies. 

#### Open field experiments

Land was cleared of weeds after which beds were raised. Cabbage (cv. Oxylus) was grown from certified seed sown on a raised bed in the field. The young seedlings were protected from pest attack with mosquito-proof netting. Standard cultural and agronomic practices such as weed control, watering and earthing-up of soil to improve aeration were employed during the growing period. The experimental design of the field trials consisted of a randomised complete block design with seven treatments and four replications. Well decomposed poultry manure at the rate of 500g per plant was incorporated into the soil two weeks before seedlings were transplanted. Cabbage seedlings were transplanted at the four true leaf stage (30 days after sowing). Spacing was 0.5 x 0.5m and plots measured 1.5m x 2.5m, resulting in 24 plants per plot. A 2m-wide unplanted alley was left between each plot to avoid spray drift between adjacent plots. Treatments were extracts of *A. conyzoides, C. odorata*, *S.* nodiflora*, N. tabacum*, *R. communis*, Attack^®^ and tap water control. Plant extract preparation was as for the field cage experiments. A separate 15L capacity knapsack sprayer was used to apply each for the treatments solutions to the point of runoff including to the underside of the leaves. Applications commenced 14 and 21 days after transplanting of seedlings for minor and major rainy seasons, respectively and were re-applied weekly thereafter. There were seven and six weekly applications for minor and major rainy seasons respectively. 

Insect presence was assessed weekly on eight plants from the two innermost rows of each plot. Infestation by *B. brassicae* was scored as described for the cage plant experiment but *P. rapae* and the common natural enemy taxa, ladybird beetles (Coccinellidae) (adults and larvae) and hoverflies (Syrphidae) (larvae) were counted in situ. Samples of larvae of coccinellids and syrphids were cultured in the laboratory to the adult life stage to allow identification by comparison with labelled specimens in the insect museum of the Entomology Section, CRI. At harvest, all the plants (24) in each plot were used for yield and insect damage assessment. 

### Statistical analyses

#### Field cage experiments

Percentage *P. xylostella* mortality data were corrected using Abbott’s formula [27]. To normalise data, percentage and score values were arcsine square root and log(x+1) transformed, respectively, before analysis. The normality of the data was tested with the Shapiro-Wilk test. Back transformed means were presented in the results. Data from the cage experiments were analysed using PROC (Univariate) the general linear model procedure of Statistical Analysis System (SAS) [28]. When significant (P<0.05) differences were obtained, means were separated using the Student Newman-Keuls (SNK) test. 

#### Open field experiments

Mean weekly count data for *P. xylostella* and natural enemies and the score data for *B. brassicae* were computed. *Brevicoryne brassicae* score data were analysed using repeated measures analysis in residual maximum likelihood (REML) using treatment and week as fixed effects and plot.time as random effect. The treatment effect was partitioned into (i) water control *versus* experimental treatments (the botanical extracts and Attack^®^ pooled) and (ii) experimental treatments. Examination of the weekly pest and natural enemy numbers showed many zeros so these data were analysed using generalised linear mixed model (GLMM) with a Poisson distribution and fixed and random terms as specified above for *B. brassicae* data. Mean cabbage head weight was analysed using ANOVA and SNK test. The proportion of cabbage heads in each plot with Lepidoptera damage were analysed using generalised linear model with a binomial distribution and logit link. All analyses were carried out in Genstat V15.

## Results

### First trophic level: plant yield and damage

In the major rainy season open field experiment *S. nodiflora*, *C. odorata* and *N. tabacum* as well as Attack^®^ had significantly (df 6; F=18.08; p<0.01) higher head weights than the water control whilst the last two mentioned botanical treatments were superior to the conventional insecticide (Table 1). For proportional damage level, all botanical treatments and Attack^®^ performed significantly (df=6,21; χ^2^=5.87, p=0.001) better than water though *A. conyzoides* and *C. odorata* were not as effective as the conventional insecticide.

**Table 1:** Effect of plant extracts and synthetic insecticides on cabbage head weight and *Lepidoptera* damage in field experiments at Kumasi, Ghana.

Treatment	Major season head weight (kg)	Minor season head weight (kg)	Minor season damaged heads (proportion)^1^
*A. conyzoides*	0.44a	0.35b	0.156b
*C. odorata*	0.70c	0.36b	0.135b
*S. nodiflora*	0.53b	0.36b	0.115ab
*N. tabacum*	0.67c	0.40b	0.125ab
*R. communis*	0.41a	0.37b	0.125ab
Attack^®^	0.56b	0.38b	0.083a
Tap water	0.35a	0.23a	0.219c
*P*	<0.001	0.006	0.001

Means within a column with different letters differ significantly (*P* < 0.05)

1 Major season proportional damage did not differ significantly between treatments (df=6,21; χ^2^=2.56, p=0.051).

In the minor rainy season experiment, botanical treatments and the Attack^®^ did not differ significantly in terms of head weight and all performed significantly (df=6; F=4.46; p<0.006) better than the water control. In the major season, when head weights were higher, the proportion of heads damaged did not differ significantly (df=6,21; χ^2^=2.56, p=0.051) between treatments (Table 1).

### Second trophic level: herbivore dynamics

#### Field cage experiments

All of the botanicals were as effective as Attack^®^ in reducing numbers of *P. xylostella* while detergent solution and tap water were less effective (Table 2). All the botanical treatments and Attack^®^ significantly reduced the *B. brassicae* score more than Lambda super^®^, 0.1% Sunlight^®^ detergent solution and tap water in the cage experiment. 

**Table 2:** Effect of plant extracts and synthetic insecticides on mean (±SE) percentage reduction of *Plutella xylostella* numbers and *Brevicoryne brassicae* infestation score (0 = absent to 5 = large continuous colonies) in field cage experiments at Kumasi, Ghana.

Treatment	*P. xylostella*	*B. brassicae*
*A. conyzoides*	100 ± 0.00 a	0.17 ± 0.17b
*C. odorata*	100 ± 0.00 a	0.17 ± 0.17b
*S. nodiflora*	93 ± 0.06a	0.00 ± 0.00 b
*C. frutescens*	93 ± 0.06 a	0.33 ± 0.33b
*N. tabacum*	93 ± 0.06 a	0.00 ± 0.00 b
*C. sophera*	66 ± 0.17ab	0.00 ± 0.00b
*J. curcas*	66 ± 0.08ab	0.00 ± 0.00 b
*R. communis*	85 ± 0.07 a	0.00 ± 0.00 b
*O. gratissimum*	89 ± 0.05a	0.17 ± 0.017b
Lambda super^®^	51 ± 0.11 b	2.33 ± 0.37 a
Attack^®^	100 ± 0.00a	0.00 ± 0.00 b
0.1% detergent solution	21 ± 0.11 c	2.33 ± 0.60a
Tap water	6 ± 0.06 c	3.00 ± 0.50 a

Means within the same column with different letters are significantly (*P* < 0.05) different from each other

#### Open field experiments

**Figure 1:**
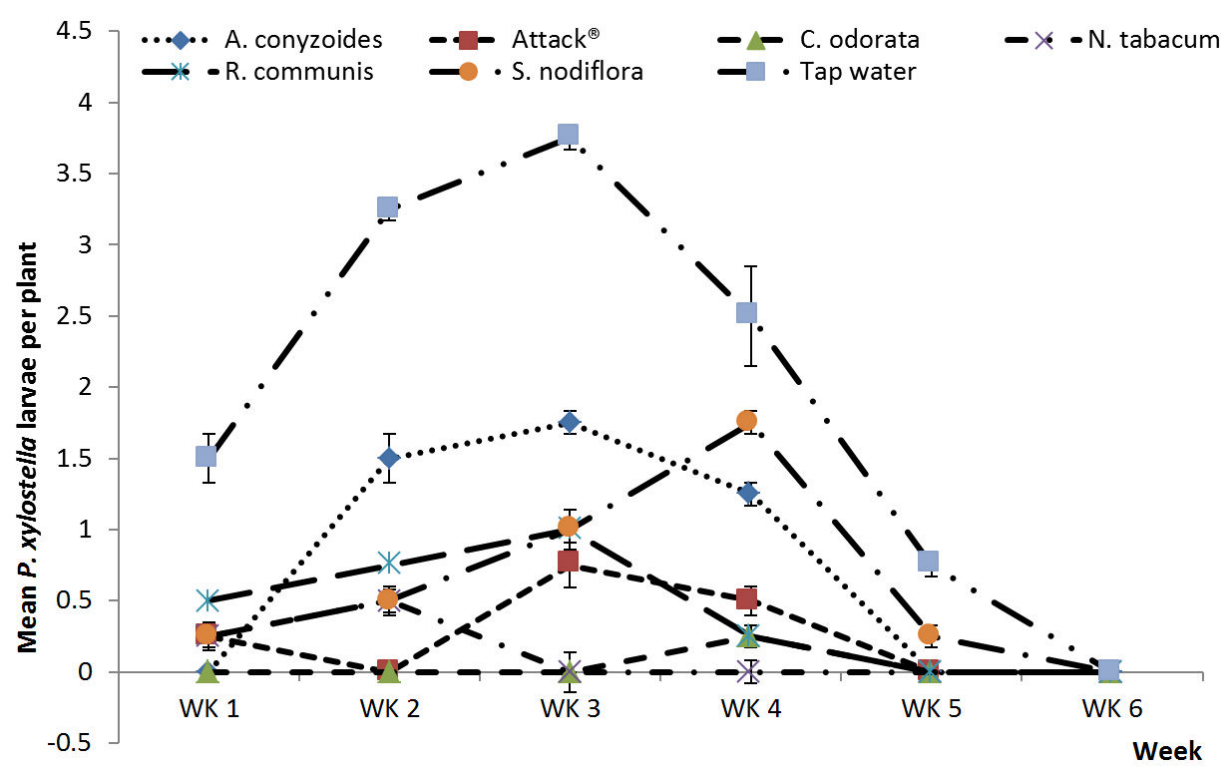
Plant extracts on *P. xylostella*, major season. Effects of plant extracts and synthetic insecticide on mean (±SE) *Plutella xylostella* count in a field experiment during the major rainy season, 2012 at Kumasi, Ghana.

**Figure 2:**
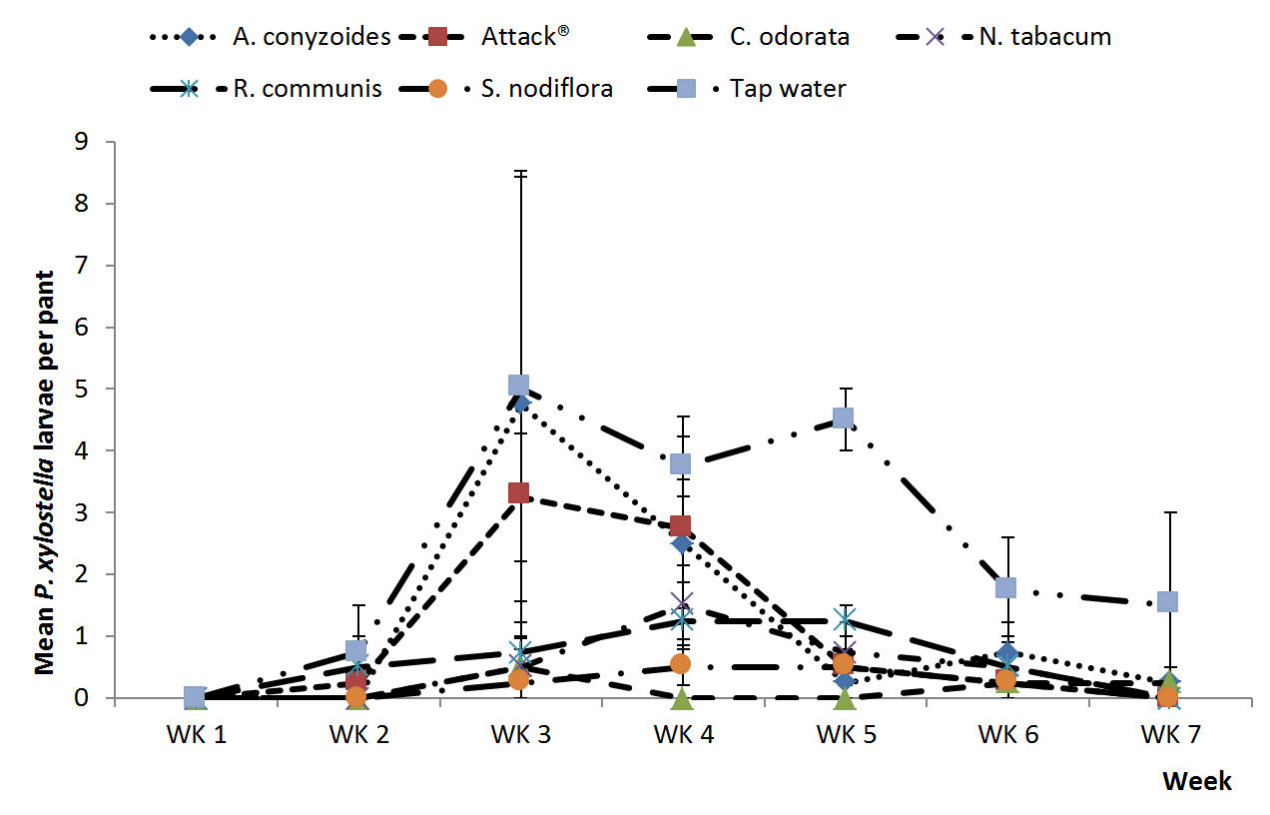
Plant extracts on *P. xylostella*, minor season. Effects of plant extracts and synthetic insecticide on mean (±SE) *Plutella xylostella* count in a field experiment during the minor rainy season, 2012 at Kumasi, Ghana.

The five botanical treatments and Attack^®^ had significantly lower overall numbers of *P. xylostella* compared to the water control during the major season (df=1,154; F=50.67; p<0.001) (Figure 1). The five botanicals and Attack^®^ differed significantly (df=5,154; F=3.27; p=0.008) with *C. odorata* giving levels of *P. xylostella* suppression greater than Attack^®^ whilst the other botanicals treatments were comparable in efficacy to Attack^®^. Results for *P. xylostella* were similar in the minor season (Figure 2). The five botanical treatments and Attack^®^ had significantly lower overall numbers of *P. xylostella* compared to the water control (df=1,179; F=21.49; p<0.001). The five botanicals and Attack^®^ differed significantly (df=5,179; F=2.51; p=0.032) with *C. odorata* and *S. nodiflora* giving levels of *P. xylostella* suppression greater than Attack^®^ whilst the other botanicals treatments were comparable in efficacy to Attack^®^.

**Figure 3:**
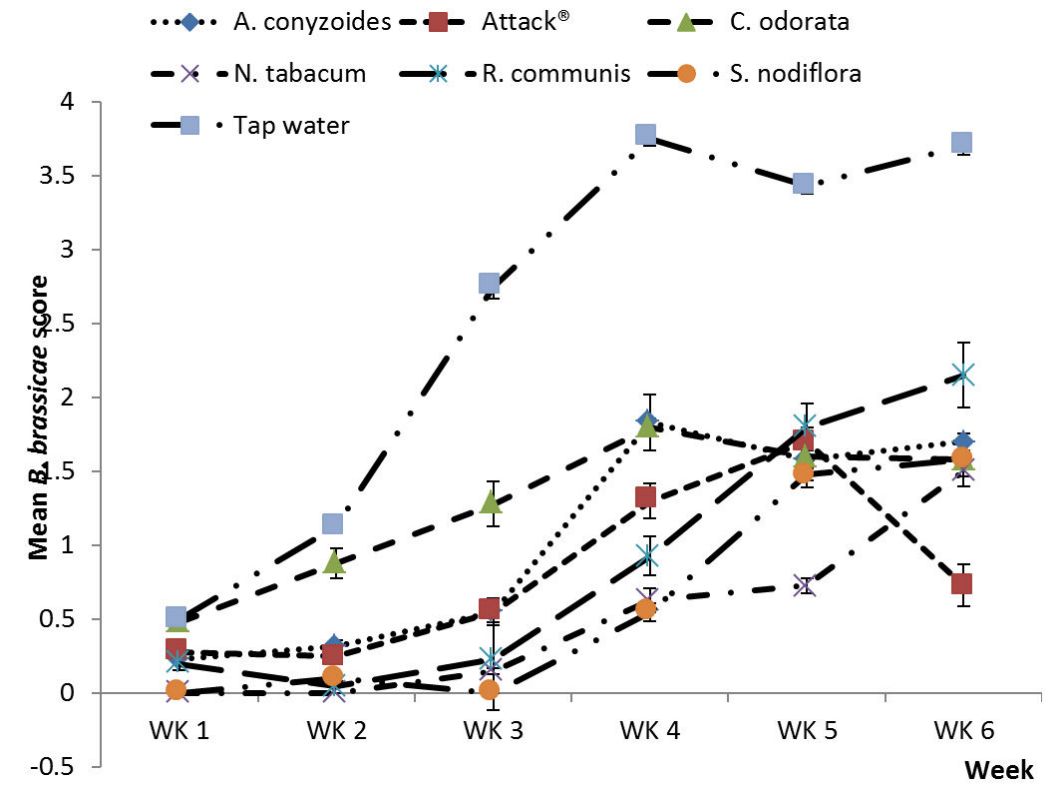
Plant extracts on *B. brassicae*, major season. Effects of plant extracts and synthetic insecticide on mean (±SE) *Brevicoryne brassicae* score in a field experiment during the major rainy season, 2012 at Kumasi, Ghana.

**Figure 4:**
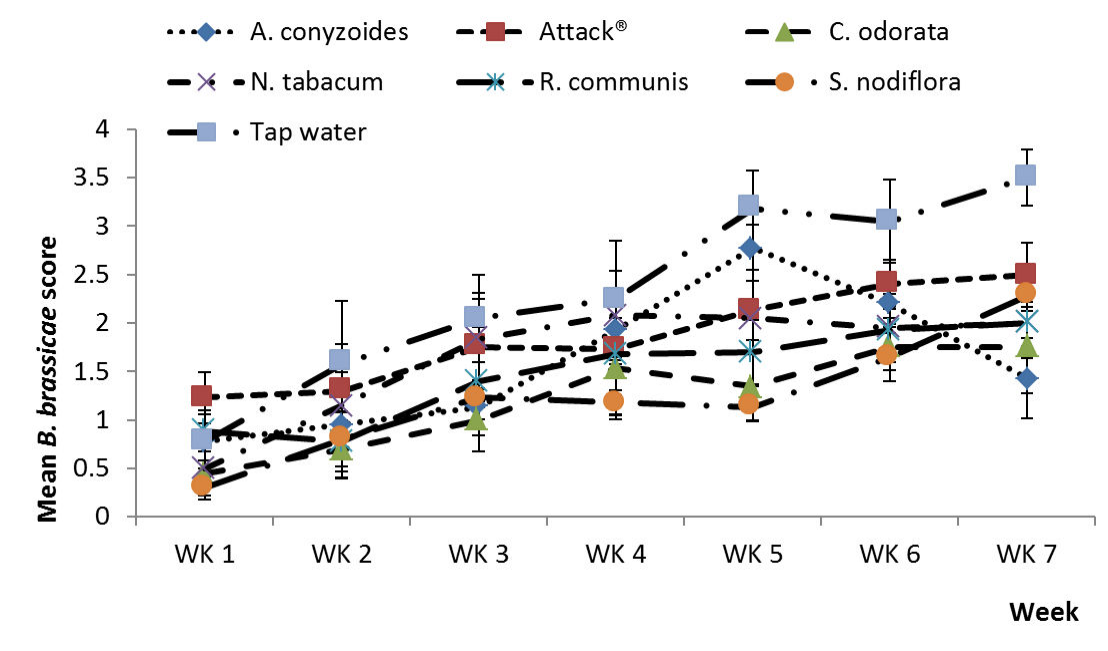
Plant extracts on *B. brassicae*, minor season. Effects of plant extracts and synthetic insecticide on mean (±SE) score of *Brevicoryne brassicae* score in a field experiment during the minor rainy season, 2012 at Kumasi, Ghana.

*Brevicoryne brassicae* infestation scores in the major season were significantly lower in the five botanical treatments and Attack^®^ compared to the water control (df=1,28; F=67.99; p<0.001) (Figure 3). Infestation levels in the water control were particularly high in the early-midseason and this is reflected in a statistically significant control.week interaction (df=1,80.7; F=16.84; p<0.001). In the minor season, *B. brassicae* infestation was significantly lower in the five botanical treatments and Attack^®^ compared to the water control (df=1,30.1; F=10.25; p<0.001) (Figure 4). Infestation levels in the water control were highest in the mid-late season and this was reflected in a statistically significant control.week interaction (df=1, 94.5; F=5.78; p<0.018). 

### Third trophic level: natural enemy dynamics

Numbers of ladybird beetles, predominantly *Coccinella magnifica* (Coleoptera: Coccinellidae), in the major season were significantly less numerous in the five botanical treatments and Attack^®^ than in the water control (df=1,154; F=15.5; p<0.001). The five botanicals and Attack^®^ differed significantly (df=5,154; F=6.39; p=0.001) with all botanicals having significantly higher coccinellid numbers than the Attack^®^ treatment and significant separation of the botanical treatments with *A. conyzoides* having the highest predator counts (Table 3). 

**Table 3:** Effect of plant extracts and synthetic insecticide on mean numbers of natural enemies per plot in a field experiment during the major rainy season, 2012 at Kumasi, Ghana.

Treatment	Ladybirds (Coccinellidae)	Hoverflies (Syrphidae)	Spiders (Araneae)
*A. conyzoides*	2.59 b	2.63 ab	0.83 ab
*C. odorata*	1.30 e	2.29 c	0.70 b
*S. nodiflora*	1.63 d	2.76 a	1.20 a
*N. tabacum*	2.25 c	2.17 c	0.83 ab
*R. communis*	2.01 c	2.73 a	0.83 ab
Attack^®^	0.49f	0.83 d	0.11 c
Tap water	3.22a	2.53 b	1.14 a
*P*	<0.001	<0.001	0.01

(Means are back transformed after analysis by generalised linear mixed model analysis). Means within a column with different letters differ significantly (*P* < 0.05)

Numbers of hoverflies, predominantly *Episyrphus balteatus* (Diptera: Syrphidae), in the major season did not differ significantly between the five botanical treatments and Attack^®^ compared to the water control (df=1,154; F=0.41; p=0.521). The five botanicals and Attack^®^ did differ significantly (df=5,154; F=5.11; p<0.001) with all botanicals having significantly higher hoverfly numbers than the Attack^®^ treatment. Of the botanical treatments, *A. conyzoides*, *S.* nodiflora and *R. communis* had significantly higher numbers of this predator taxon than did *C. odorata* and *N. tabacum* (Table 3). 

Numbers of spiders (Araneae) in the major season did not differ significantly between the five botanical treatments and Attack^®^ compared to the water control (df=1,151; F=1.63; p=0.204. The five botanicals and Attack^®^ did differ significantly (df=5,151; F=3.16; p=0.01) with all botanicals having significantly higher spider numbers than the Attack^®^ treatment though *C. odorata* had lower numbers of this predator taxon than did *S. nodiflora*, the botanical treatment in which spiders were most numerous (Table 3). 

Numbers of all three natural enemy taxa were lower in the minor season than in the major season and did not exhibit significant treatment differences. 

## Discussion

The effectiveness of the botanical treatments in this study was generally equivalent to that of conventional, synthetic insecticide Attack^®^ in managing *P. xylostella* and *B. brassicae*. Poor control of *B. brassicae* was observed for Lambda Super^®^ in the field cage experiment. A similar observation was made for Bossmate^®^ (lambda-cyhalothrin) which failed to control *B. brassicae* in a field experiment in Ghana resulting in a reduced yield of plots sprayed with Bossmate^®^ compared to plots sprayed with garlic, chili pepper and Attack^®^ [23] The lack of control by this conventional insecticide was attributed to resistance in the aphid population and this is likely to have also been the case in the present study. The efficacy of the botanical treatments against *B. brassicae* and *P. xylostella* supports the findings of other previous studies of these and related pests. For example, extracts of *Azadirachta* and *Melia azedarach* have been successfully used to control *B. brassicae* [29,30]. Similarly, leaf extracts of *R. communis* and *S. nodiflora* as well as *J. curcas*, have been used to manage the lepidopterans *Spodoptera litura* and *Achaea janata* [16,20,31]. Another treatment used in the present study, *N. tabacum* has previously been reported to have been used to manage both *P. xylostella* and *B. brassicae* [32]. 

In the current study, *P. xylostella* and *B. brassicae* populations were effectively suppressed with the evaluated botanicals and Attack^®^. Insect pests of brassicas such as the *P. xylostella* have been managed successfully with botanical insecticides [33-35]. 

Conversely, *P. xylostella* has been difficult to control with synthetic insecticides in many regions of the world because of the development of insecticide resistance [32,33,35,36]. *Plutella xylostella* is reported to be the first crop pest to have developed resistance against dichlorodiphenyltrichloroethane (DDT) and the first to develop resistance to *Bacillus thuringiensis* (*Bt*) insecticides [33,37,38] thus, any botanical that is able to offer a significant control will be considered valuable. In Brazil [39], reported that it is normal for farmers to apply between 15-20 insecticide sprays within a cropping season with at least three applications in a week, without success, in an effort to reduce yield losses caused by *P. xylostella*. The ability of the botanicals to significantly manage the populations of *P. xylostella*, as well as *B. brassicae*, in this study is an indication of their potential usefulness in integrated pest management (IPM) of cabbage, especially for resource-limited farmers. 

The *numbers* of *P. xylostella* and *B. brassicae* were generally higher in the minor rainy season compared with the major rain season. This may be due to the fact that the amount of rainfall and the frequency is usually high in the major rainy season and this could disrupt the reproduction of the insects or dislodge them from plants. 

There are suggestions that natural enemies should be the first consideration in any pest management intervention [40]. Any integrated approach to pest management must thus, be compatible with natural enemy conservation. Emamectin benzoate (Attack^®^) is regarded as a novel semi-synthetic derivative of the natural product abamectin in the avermectin family and is known to be effective against a wide range of pests [41]. However, in this study, Attack^®^ caused a significant reduction in the numbers of ladybirds, hoverflies and spiders. Both of the pests monitored in the present study are attacked by a wide range of natural enemies when free from the effects of insecticide use [42]. Until the early 1960s when large scale application of synthetic insecticides was introduced to commercial vegetable farming, *P. xylostella* was not a major pest in China [43]. The use of pesticides that are harmful to the third trophic level need to be minimised in favour of less harmful plant protection compounds to allow biological control to play a role in integrated pest management [44]. In particular, an active natural enemy fauna can reduce the frequency with which insecticides applications are required and kill survivors of insecticide application to prevent the development of resistance in the pest population. Not all botanical insecticides are able to play this role. Some such as pyrethrum are broad spectrum in nature [22,45]. Those used in the present study appeared much less toxic to the natural enemies that were common in the study site, ladybirds, hoverflies and spiders, than was the conventional insecticide, Attack^®^. These members of the third trophic level had densities on the botanical extract treated plants similar to the water control though *C. odorata* gave consistently over all three natural enemy taxa lower than the best performing botanicals. Given the relatively small scale of the experiments, with plots separated by just 2m, the Attack^®^ plots would have been subject to potential recolonisation by natural enemies moving from neighbouring plots. That numbers of predators were consistently low in the Attack^®^ treatment despite this phenomenon highlights the negative impact of such broad spectrum insecticides on biological control. The consistently low numbers of natural enemies in plots sprayed with Attack^®^ could also result from the possibility that this pesticide repelled them rather than being due to mortality.

Notwithstanding the higher numbers of natural enemies in the water control, this treatment was heavily attacked by both *B. brassicae* and *P. xylostella* and had poor first trophic level performance. These metrics show that effective biological control by natural enemies was not achieved in this system solely by withholding insecticide and that botanical insecticides have an important role in pest suppression. 

The compatibility of the botanical treatments in this field study with common natural enemies is in broad agreement with earlier work on some other botanical insecticides. No negative impact was found on the parasitoids, *Cotesia plutellae* and *Diadromus collaris* when *M. azedarach* and *A. indica* extracts were applied against *P. xylostella* [35]. Reports by [23] and [9] indicate that effective control of cabbage pests with garlic and chili pepper at 10, 20 and 30g/L of water was achieved whilst beneficial insects were preserved. However [23], cautioned that, at higher concentrations, botanicals could possibly have some detrimental effect on natural enemy populations because their numbers reduced with increasing concentration of garlic and chili pepper extracts. 

Effects at the second and third tropic levels are complemented in the present study by marked effects at the first trophic level, an important observation in the use of simply prepared readily available botanical insecticides. 

This study has revealed that extracts of *C. sophera, O. gratissimum, N. tabacum*, *S.* nodiflora*, R. communis, A. conyzoides, J. curcas* and *C. odorata* have good potential for use in managing pests of cabbage. Two factors make this observation particularly important for poor farmers in this region and potentially in other parts of the world. First, the plant extracts were prepared by simple mechanical means using only detergent and water. This contrasts with the often employed-organic solvent, steam distillation and other approaches used for preparing botanical insecticides. Thus, the types of treatments shown to be promising in the present study could be easily prepared by resource-limited farmers or as cottage industry enterprises in rural villages. Second, the plants themselves are cheap and readily available to farmers. Many grow as weeds so are immediately available at no cost, whilst chili and tobacco are widely grown so offering scope for the use of crop residues, damaged or excess materials in plant protection. 

Cabbage is often eaten raw and managing its pests with botanical insecticides will contribute markedly to food safety. The beneficial effects of the botanical extracts at the first, second and third trophic levels provide justification for further studies of such plant protection products including the range of pests to which they are active and the measurement of sub-lethal effects on various natural enemy taxa. The labour intensive nature of the preparation of sprays based on these plant extracts is not likely to be a constraint on their use by smallholder families but likely to be an impediment to usage on large scale commercial farms. This obstacle may be overcome, however, if efforts are made to commercially extract the active components and package them for sale.
