# Correction: Tri-Trophic Insecticidal Effects of African Plants against Cabbage Pests

**DOI:** 10.1371/annotation/f0351003-b6f8-4249-ace5-bcd84dead916

**Published:** 2013-11-15

**Authors:** Blankson W. Amoabeng, Geoff M. Gurr, Catherine W. Gitau, Helen I. Nicol, Louis Munyakazi, Philip C. Stevenson

The name of the last author was given incorrectly. The correct name is: Philip C. Stevenson. 

